# Housing Status and Healthcare Utilization in People Presenting With Seizure

**DOI:** 10.1177/19418744251321877

**Published:** 2025-02-13

**Authors:** Sandeepa S. Mullady, Andrew J. Wood, Elan L. Guterman, Nicole Rosendale

**Affiliations:** 1 Memory Divisions, Department of Neurology, 6429 Stanford University, San Francisco, CA, USA; 2 Department of Neurology, 8785 University of California San Francisco, San Francisco, CA, USA; 3 Weill Institute for Neurosciences, 8785 University of California San Francisco, San Francisco, CA, USA; 4 Philip R. Lee Institute for Health Policy Studies, 8785 University of California San Francisco, San Francisco, CA, USA

**Keywords:** homelessness, seizure, healthcare utilization, health equity

## Abstract

**Objective:**

To examine the association between housing status and healthcare utilization in individuals presenting with seizure.

**Methods:**

We performed a retrospective cross-sectional analysis of all adults (age >18) presenting to a public hospital emergency department with seizures, defined by ICD-9/10 codes, between 1/1/2016 and 8/03/2019. They were categorized by housing status (people experiencing homelessness [PEH], people with housing). Healthcare utilization outcomes were 30-day re-visit to acute care, discharge disposition, and hospital length of stay for those admitted. We used multivariable linear and logistic regression models adjusting for age, comorbidities, and insurance status.

**Results:**

There were 6483 individuals (2092 [32.3%] PEH). Compared to people with housing, PEH were younger (48.2 vs 50.9, *P* < .0001), more likely to be a person of color (80.9 vs 75.1%, *P* < .0001), and have Medicaid (51.4% vs 42.9%, *P* < .0001). People with housing had a higher prevalence of admission to the intensive care unit (3.6% vs 1.8%, *P* < .0001). After adjustment, admitted PEH had higher odds of 30-day re-visit (adjusted odds ratio [aOR] 1.87, 95% confidence interval [CI] 1.58, 2.21), shorter length of stay (coef Β-12.87, 95% CI: −22.62, −3.11), and lower odds of being discharged to a facility (aOR 0.37, 95% CI: .26, .55) compared to people with housing.

**Conclusion and Relevance:**

PEH with seizures had increased healthcare utilization. Further analysis, including imaging findings, anti-seizure medications prescribed, and presumed etiology, is needed to understand the drivers of healthcare utilization and identify appropriate interventions.

## Introduction

Homelessness is an epidemic, with an estimated 582,462 people experiencing homelessness on a single night in the US.^1^ More than half of people experiencing homelessness (PEH) in the U.S. reside in California.^2^ PEH have high rates of healthcare utilization in acute care settings.^3^ Prior research suggests that seizure is a common indication for admission in PEH,^4^ however, little is known about healthcare utilization in this population.

The objective of this study was to assess the association between housing status and healthcare utilization in a sample of people presenting to the emergency department after seizure.

## Methods

We performed a retrospective cross-sectional analysis of all adult patients (aged 18 years or older) who presented to the emergency department (ED), regardless of subsequent admission, at Zuckerberg San Francisco General Hospital (ZSFG) with a primary presentation diagnosis of seizure, defined by ICD-9 (345.x, 780.3, 333) and ICD-10 codes (G40, G41, R56), between January 1, 2016 and August 3, 2019. Due to conversion to another electronic health record (EHR) system, more recent data were not available.

Participant sociodemographic characteristics, including housing status, were obtained as they were coded in the EHR. Race was categorized as Asian, Black, White, or another race. Comorbidities (substance use, traumatic brain injury, stroke, infection, psychiatric conditions, non-epileptic seizures, dementia) were obtained using ICD9/10 codes. Specifically, for non-epileptic seizures, we used ICD-9 codes 300.1 and 300.11 (Dissociative, conversion, factitious; conversion) and ICD-10 code F44.5 (Dissociative convulsions) to avoid incorrectly identifying epileptic seizures as non-epileptic.^5^ Healthcare resource use (CT imaging, MR imaging, and EEG ordered on presentation date), and markers of illness severity (intensive care unit [ICU] admission, intubation) were obtained using billing codes. Primary outcomes were 30-day re-visit, length of stay, and discharge disposition in all those presenting to the ED. The 30-day re-visit variable was categorized as a binary (yes/no) variable based on the date between the first presentation and subsequent presentation, either to the ED or as a hospital readmission. Discharge disposition was dichotomized to self-care vs facility due to small sample sizes within individual categories. All patients at ZSGF receive social work and case management services, and the vast majority (regardless of immigration status) are insured under a Medicare or Medicaid insurance plan; thus, these variables were not included in the analysis.

We included those with complete housing information in the final analysis (n = 928 [12.5%] missing). We divided the population based on housing status and performed univariate analyses using 2 sample t-tests for continuous variables and Pearson’s chi-squared test for categorical variables. We assessed the association between homelessness and 30-day re-visits, length of stay, and discharge disposition using multivariable linear and logistic regressions, adjusting for age, comorbidities including psychiatric condition, dementia, stroke, infection, substance use, and traumatic brain injury, and insurance status. To investigate the role of race in the association between housing status and healthcare utilization, we performed regression models adjusting for the same independent variables adding an interaction term for race and housing status.

Statistical significance was set at α < .05 for both the univariate and multivariate analyses. Statistical analyses were performed with Stata (version 17, StataCorp, College Station, TX).

This study was approved by the institutional review board of University of California, San Francisco (certificate 16-19911) and was exempt from the need for informed consent as no direct participant contact was made. It was reported in alignment with The Strengthening the Reporting of Observational Studies in Epidemiology (STROBE) guidelines (Supplement 1).

## Results

There were 6483 individuals who presented to the ED with seizure with complete housing status (2092, 32.3% PEH). PEH were younger (48.2 vs 50.9, *P* < .0001), more likely to be people of color (80.9 vs 75.1%, *P* < .0001), and more likely to be insured by Medicaid (51.4% vs 42.9%, *P* < .0001) (Table 1). PEH were less likely to be admitted (54% vs 68.5%, *P* < .0001; Table 1). Comorbidities differed between the groups: PEH had higher likelihood of substance use (86.3% vs 60.6%, *P* < .0001), higher likelihood of traumatic brain injury (24.0% vs 17.4%, *P* < .001), lower likelihood of stroke (5.5% vs 22.1%, *P* < .0001), and lower likelihood of non-epileptic seizures (4.5% vs 8.5%, *P* < .0001; Table 1).

**Table 1.** Characteristics of Participants With Seizure, Stratified by Housing Status.

N	Homeless	Housed	*P*-Value
	2092	4391	
Patient demographics			
Age, mean (SD)	48.2 (12.0)	50.9 (17.9)	<.0001
Sex, N (%)			<.0001
Female	370 (17.8%)	1562 (36.2%)	
Male	1713 (82.2%)	2758 (63.8%)	
Missing	9 (.4%)	71 (1.6%)	
Race, N (%)			<.0001
Asian	50 (2.4%)	436 (10.0%)	
Black	777 (37.3%)	1518 (34.8%)	
White	398 (19.1%)	1084 (24.9%)	
Another race	860 (41.2%)	1320 (30.3%)	
Missing	7 (.3%)	33 (.8%)	
Ethnicity, N (%)			<.0001
Hispanic or latine	338 (16.8%)	950 (22.0%)	
Not hispanic or latine	1670 (83.2%)	3378 (78.0%)	
Missing	84 (4.0%)	63 (1.4%)	
Insurance, N (%)			<.0001
Medicare	291 (13.9%)	722 (16.4%)	
Medicaid	1076 (51.4%)	1883 (42.9%)	
Enrolled in part C	26 (1.2%)	19 (.4%)	
Medicare + Medicaid	7 (.3%)	28 (.6%)	
Uninsured	46 (2.2%)	78 (1.8%)	
Missing	646 (30.9%)	1661 (37.8%)	
Clinical characteristics, N(%)			
Inpatient admission			<.0001
Not admitted/emergency room only	1433 (68.5%)	2373 (54.0%)	
Admitted/inpatient	659 (31.5%)	2018 (46.0%)	
Admission source, N (%)			<.0001
Long term care facility	2 (.1%)	25 (.6%)	
Place of temporary or permanent residence	574 (27.4%)	1560 (35.5%)	
Psych emergency services	6 (.3%)	15 (.3%)	
Skilled nursing facility (SNF)	4 (.2%)	83 (1.9%)	
Other^a^	40 (1.9%)	78 (1.8%)	
Missing	1433 (68.5%)	2376 (54.1%)	
Comorbidities, N (%)			
Infection	15 (.8%)	70 (2.1%)	.0002
Dementia	469 (24.2%)	1016 (30.7%)	<.0001
Non-epileptic seizures	87 (4.5%)	282 (8.5%)	<0.0001
Psychiatric	1622 (83.7%)	2644 (80.0%)	.001
Stroke	106 (5.5%)	731 (22.1%)	<.0001
Substance abuse	1673 (86.3%)	2004 (60.6%)	<.0001
Traumatic brain injury	465 (24.0%)	575 (17.4%)	<.0001
Presentation information, N(%)			
Intensive care unit admission	37 (1.8%)	159 (3.6%)	<.0001
Intubation	1 (<1%)	2 (<1%)	.97
EEG ordered on presentation	8 (.4%)	33 (.8%)	.0796
CT order on presentation date	634 (31.4%)	1481 (34.0%)	.0448
MRI order on presentation date	25 (1.2%)	171 (3.9%)	<.0001
Admitted length of stay, median (IQR)	3.0 (2.0, 8.0)	4.0 (2.0, 11.0)	.0002
30-day re-visit	572 (27.8%)	521 (12.2%)	<.0001
Discharge destination, N (%)			<.0001
AMA	80 (12.2%)	103 (5.2%)	
Dead	21 (3.2%)	106 (5.3%)	
Self-care	460 (70.0%)	1186 (59.4%)	
SNF/rehabilitation facility	29 (4.4%)	270 (13.5%)	
Transfer to acute care facility	29 (4.4%)	258 (12.9%)	
Other^b^	38 (5.8%)	72 (3.6%)	

^a^Other includes offsite and onsite clinic, correctional facility, acute rehab, and hospice.

^b^Other includes forensic facility, terminal care, hospice, and foster home.

PEH also had lower likelihood of ICU admission (3.6% vs 1.8% *P* < .0001) and undergoing MRI on presentation to the acute care setting (3.9% vs 1.2%, *P* < .0001).

Compared to those with housing, PEH had higher odds of a 30-day re-visit (adjusted odds ratio [aOR] 1.87, 95% CI: 1.58, 2.21), shorter length of stay (coef −12.87, 95% CI: −22.62, −3.11) and lower odds of being discharged to a facility (aOR 0.37, 95% CI: .26, .55) after adjustment (Table 2). There was no significant interaction of race with housing status on these associations.

**Table 2.** Association Between 30-Day Re-Visits, Length of Stay, and Discharge With Housing Status in People Presenting With Seizure.

Homeless	Unadjusted	Age and Comorbidity Adjusted	Age, Comorbidity, and Insurance Adjusted
30-day re-visit, OR (95% CI); *P*-val			
N	6339	5135	3691
	2.78 (2.43, 3.17); .0000	2.04 (1.76, 2.36); .0000	1.87 (1.58, 2.21); .0000
Admitted length of stay, coef (95% CI); *P*-val			
N	2677	2212	1543
	−11.87 (−17.90, −5.84); .0001	−9.69 (−16.96, −2.41); .0091	−12.87 (−22.62, −3.11); .0098
Discharge to another facility vs Self-Care, OR (95% CI); *P*-val			
N	2415	1987	1402
	.26 (.20, .35); .0000	.40 (.29, .55); .0000	.37 (.26, .55); .0000

## Discussion

In this cross-sectional analysis of people presenting with seizure to an urban public hospital, PEH had higher odds of 30-day re-visits, shorter lengths of stay, and lower odds of discharge to a facility. In this hospital system, all PEH are evaluated by social work and case management services and there are robust resources available for patient assistance. Despite these resources, disparities remain.

The increased odds of 30-day re-visits amongst PEH in our study may be related to a lack of medication access or limited seizure health literacy amongst PEH. In a national study examining epilepsy readmissions, repeat convulsion was the most cited reason for return to hospital suggesting the importance of medication titration or adherence.^6,7^ Alcohol use and psychosis were found to increase risk of readmission.^6,7^ There was a higher prevalence of psychiatric conditions and substance use in PEH in our study, which may have contributed to increased re-visit rate.

Our finding that PEH had shorter lengths of stay is different than other studies that show increased hospital lengths of stay among PEH.^8^ The prevalence of patient-directed discharge was higher in PEH in our sample and may partially explain these findings. Limitations in city-wide shelter resources may encourage individuals to join shelter waitlists that are filled on a first-come-first basis rather than await further work-up in the hospital setting. The 2023 California Statewide Study of People Experiencing Homelessness found that 41% of individuals who desired shelter were unable to do so.^9^

The prevalence of dementia itself was found to be lower in our study population; in general, cognitive impairment is higher amongst PEH.^10^ Seizures itself can contribute to cognitive decline in PEH, and this may impact a person’s understanding of the goals of the hospitalization or willingness to remain admitted.^11^ Shorter lengths of stay in PEH may contribute to incomplete titration of anti-seizure medication that result in increased re-visits. Patient acuity, measured through intubation and ICU rates, were lower amongst PEH and may be driving lengths of stay as well.

Prior interventions have focused on access to primary care. Our study makes a case for the development of pathways to improve access to neurologic care. One such previously established pathway involved automatic consultation of epilepsy services for all PEH presenting with seizure etiology and led to improved dissemination of information and integration of care to the outpatient setting.^12^ Integration of primary care services into homeless shelters has been shown to reduce barriers to access and increase preventative care.^13^ Integrating neurology into underserved settings such as street medicine clinics and federally qualified healthcare centers may similarly improve access. Transforming neurology from a subspeciality care service to a primary care model would allow for preventative neurologic care and potential unburdening of acute care systems.

Limitations include our mechanism of coding homelessness, which is based on demographic data in the EHR. Our designation of PEH was not sensitive enough to capture all those that may be unhoused, which biased the result towards null. Identifying other sociodemographics, such as primary language or immigration status, may elucidate other barriers to access, however, individuals in California have access to healthcare insurance regardless of immigration status. Despite the high rates of coverage in PEH in our study, disparities in healthcare utilization remain. We were not able to extract imaging results, medications, and presumed etiology of seizures, which may ascertain predictors in healthcare utilization, including differences in seizure severity and access to appropriately dosed medications. Additionally, this analysis is from a single center and, therefore, may not be generalizable to the larger population.

## Conclusions

PEH with seizures have high healthcare utilization despite potentially lower clinical acuity. Our study suggests that these disparities exist even within a healthcare system with robust social work support. Further research better characterizing the reasons for re-visits in PEH will be helpful in devising systemic solutions.

## Supplemental Material


Supplemental Material - Housing Status and Healthcare Utilization in People Presenting With Seizure
Supplemental Material for Housing Status and Healthcare Utilization in People Presenting With Seizure by Sandeepa S. Mullady, Andrew J. Wood, Elan L. Guterman, and Nicole Rosendale in Journal of the Neurohospitalist.
